# Optimizing health care delivery by adapting diagnostics in a low-resource setting: The case of San Miguel Hospital, Sucumbíos, Ecuador^☆^

**DOI:** 10.1016/j.nmni.2025.101696

**Published:** 2025-12-31

**Authors:** Willemijn Johanna Catharina van Keizerswaard, Jacob van der Ende, Carolien Maria Bouwman, Maria Vanessa Dávila Campos, Martin Peter Grobusch

**Affiliations:** a Center of Tropical Medicine and Travel Medicine, Department of Infectious Diseases, Amsterdam University Medical Centres, Amsterdam Public Health-Global Health, Amsterdam Infection and Immunity, Amsterdam, the Netherlands; b Hospital San Miguel, Puerto El Carmen de Putumayo, Sucumbíos, Ecuador; c University of Tübingen, Tübingen, Germany; d Centre de Recherches Médicales en Lambaréné (CERMEL), Lambaréné, Gabon; e Masanga Hospital, Masanga, Sierra Leone; f University of Cape Town, Cape Town, South Africa

**Keywords:** Diagnostic tools, Resource-limited setting, Health systems evaluation, Disease prevalence, Indigenous health

## Abstract

**Background:**

To strategically optimize diagnostic capacity in a low-resource, rural hospital setting, we developed a systematic evaluation of diagnostic tool needs and associated costs. This local data-driven method, accounting for patient characteristics and disease prevalence, can be adapted to other contexts.

**Methods:**

A retrospective patient record analysis was conducted at San Miguel Hospital (SMH) in Sucumbíos, Ecuador, which provides outpatient and emergency care to inhabitants of the Ecuadorian and Colombian Amazon basin. Ethics approval was granted retrospectively by the Research Ethics Committee on Human Beings of the Universidad San Francisco de Quito.

Data was retrieved from electronic medical records (EMRs) of the first 796 patients seen after hospital opening. For each of the 1975 diagnoses made, patient characteristics and the presence or absence of appropriate diagnostic tools were recorded. Unavailable tools were further evaluated for accessibility within the local context.

**Results:**

Serving a population primarily of mixed and indigenous ethnicities, SMH confirmed 66 % of diagnoses using existing resources, with potassium hydroxide (KOH) fungal microscopy, chikungunya and influenza rapid tests, and access to anatomical pathology identified as the diagnostic tools offering the highest return on investment.

**Conclusions:**

Data from SMH's EMRs suggest which diagnostic tools would offer the greatest return on investment through increased diagnostic confirmation. This evaluation tool supports improved health care delivery at SMH and, with adaptation, can be applied in comparable health care settings.

**Trial registration:**

N/A.

## Highlights

We evaluated the availability and utilization of diagnostic tools in a rural hospital in Ecuadorian Amazonia using patient data. Our approach identifies high-impact diagnostic resources needed to support clinical decision-making and can be adapted to strengthen health care delivery in similar low-resource settings.

## 1 Background

The world population exceeded 8 billion people in 2022, with 3.5 billion lacking sufficient healthcare access [1]. Providing healthcare in rural areas has been a global challenge, requiring political commitment, resources, and investments. Around 10 % of global GDP is spent on healthcare [2], while low- and middle-income countries spend only 5 %. Local, context-specific initiatives are believed to play a key role in optimizing scarce health resources, improving both access and quality sustainably [2]. One such initiative is San Miguel Hospital (SMH) in the Amazon rainforest, Ecuador. Located in the canton of Putumayo, within the province of Sucumbíos, Ecuador, and bordering Colombia and Peru, SMH provides primary and emergency healthcare to one of South America's most remote regions **(Appendix –** Supplementary Fig. S1).

The area, known for high coca plantation density and ongoing guerrilla conflict, has been classified as unsafe, severely limiting healthcare access [3,4]. Environmental contamination from oil extraction in the Amazon worsens public health outcomes [5], while climate change increases the prevalence of vector-borne diseases and disrupts agriculture, threatening food security [6]. Widespread deforestation accelerates environmental degradation, worsening health challenges for indigenous populations [7].

The nearest hospital is a 4-h drive away, with a government-run medical post providing unreliable diagnostics, medicines, and vaccinations. To fill this gap, a Dutch NGO founded SMH in November 2021 [8]. Initially, only outpatient services were offered until the clinical wards opened two years later. The hospital uses an electronic patient record system and serves over 20 000 people, including indigenous communities like the Kichwa, Shuar, and Siona [9]. These populations face the greatest challenges in accessing healthcare and are more likely to contract and die from waterborne diseases like dysentery [13].

As SMH grows, data is needed to guide investments in knowledge, staff, and equipment particularly during the hospital's initial operational phase, when diagnostic planning is most impactful. This study identifies which diseases can and cannot be diagnosed with available tools and which diagnostic tool investments offer the highest return on investment, an approach that was directly used to inform early diagnostic expansion at SMH. This data is critical, as diagnostic gaps can lead to both over- and under-treatment, highly undesirable outcomes. While the specific findings are shaped by the local population and epidemiology, we aim to provide a methodology based on the assessment of need and accessibility of diagnostic tools in low-resource rural settings that can be adapted to inform locally appropriate strategies in similar healthcare contexts.

## 2 Methods

### 2.1 Setting

This retrospective patient records analysis took place in the SMH, located in the canton of Putumayo, Province of Sucumbíos, Ecuador (**Appendix** - Supplementary Fig. S1).

### 2.2 Data collection, storage and handling

Patient medical records were registered electronically from the first day of hospital operations using a self-developed hospital information system. This enabled continuous data collection for the first 796 patients **(Appendix –** Supplementary Fig. S2). Anonymized records of patients who consulted a physician between November 2021 and February 2022 were included retrospectively. The system captured demographic characteristics at registration and clinical information entered by physicians, including anamnesis, physical examination findings, diagnostic findings, diagnoses, and treatment plans. Patient data from international employees were excluded to ensure a more accurate representation of the local population. This exclusion applied specifically to international employees, as they relocated solely for work. However, patients born outside Ecuador were not excluded, as many had Ecuadorian parentage or had lived in the country long-term.

Data were securely stored on a protected server and analyzed on Chromebooks within the protected environment of Google Workspace. Data analysis was conducted using RStudio (version 2023.06.0 + 421), developed and distributed by Posit Software (Boston, MA). The analysis environment ensured data security and compliance with privacy protocols. Each consultation was entered as a separate data entry, resulting in more diagnoses than consultations. Incomplete registrations were excluded from the study (n = 28). For each diagnosis, we registered the patient's demographics, whether it was the first time presenting the issue, diagnostic tools used, whether the tools were sufficient to confirm the final diagnosis, and which tools were missing. The decision to confirm a final diagnosis was based on Dutch National Guidelines for general practitioners (Nederlands Huisartsen Genootschap (NHG)) [14], discussed with a physician when not applicable to the local context until consensus was reached. When patients provided diagnostic test results from external medical institutions, these results were considered in confirming the final diagnosis. However, the corresponding diagnostic tool was still recorded as missing in SMH, as external tests—such as a prostate-specific antigen (PSA) test—were not yet implemented within SMH's list of applied diagnostic tools. If an external test was crucial for establishing or ruling out a diagnosis, it was marked as a missing diagnostic tool in SMH. The accessibility of the diagnostic tools missing defined in Table 1 was rated by two physicians (CMB, JVDE). Their years of experience in the preparation and creation of the hospital gave them good insights into local availability of different diagnostic tools. Information such as the origin, price, technical support availability, and the likelihood of obtaining a license for use was gathered during these years. After confirmation by local staff, this information allowed for an accurate estimation of the accessibility of the diagnostic tools.

**Table 1:** Criteria used to score the accessibility of diagnostics tools in San Miguel Hospital.

Score	Accessibility	Criteria
4 =	Easy	Costs: installation <$3000
		AND/OR no to less than one week of training for medical staff
3 =	Easy-medium	Costs: installation $3000 - $10,000
		AND/OR training of medical staff ∼1 week
2 =	Medium - difficult	Costs: installation $10,000 - $50,000
		AND/OR extensive training of staff needed 4 weeks
		AND/OR in-house diagnostic harder to obtain than collaboration with other hospital
1 =	Difficult	Costs: installation > $50,000
		AND/OR training of staff unfeasible or >2 months
		AND/OR in-house diagnostic harder to obtain than collaboration with other hospital
		AND/OR other healthcare providers facilitating the service in the region

Costs are provided in United States Dollar (USD$).

Rated by two physicians with extensive experience in establishing the hospital leveraged their knowledge of local diagnostic tool availability, costs, licensing, and technical support, and, after validation by local staff.

**Fig. 1:**
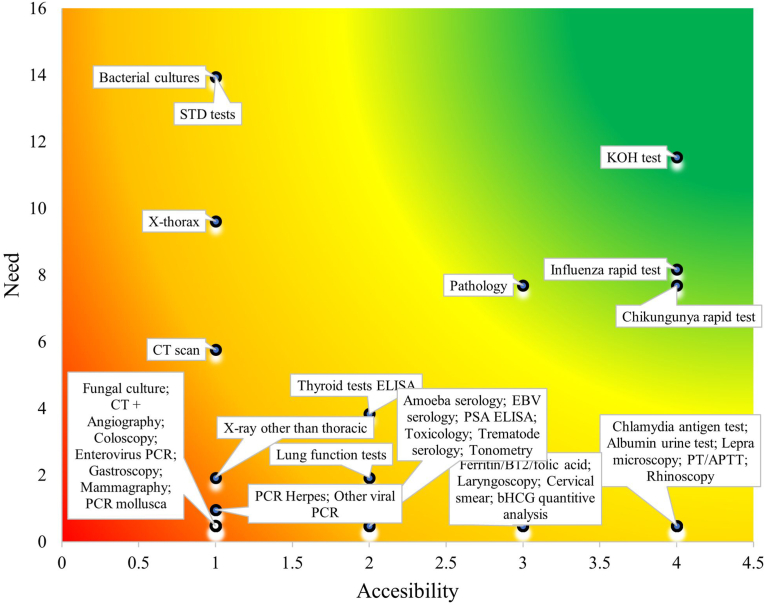
The need and accessibility of diagnostic tools in San Miguel Hospital. The need is defined by the frequency with which a diagnostic tool was reported missing between November 2021 and February 2022. The accessibility is defined on a scale from 1 to 4, 1 corresponding to the tools that are the most difficult to obtain and 4 to the tools most easily acquired in the province Sucumbíos, Ecuador.

Fig. 1 was created using Microsoft Excel by plotting diagnostic tests according to two continuous variables: Need (y-axis) and Accessibility (x-axis). Each diagnostic tool is represented by a point placed according to its respective scores for these dimensions, based on data collected from healthcare professionals and resource availability assessments.

The continuous color gradient background - from red/orange (low accessibility and high need) through yellow (moderate accessibility and need) to green (high accessibility and lower need) - was generated by applying a custom two-dimensional conditional formatting technique in Excel. This was done by overlaying a smooth gradient fill across the plot area, visually emphasizing the prioritization of diagnostic tools based on combined accessibility and need metrics.

## 3 Results

### 3.1 Patient demographics and disease prevalence

Ecuadorians are the main population served by the hospital (**Appendix –** Supplementary Table S1). Ninety percent (n = 714/796) of the patients represented Mestizos; a mix of Indigenous and European ethnicity.; Most patients lived within 5 km (3.1 miles) from the hospital (474/796; 60 %). The majority were female (501/796; 63 %). In 30 of the 796 cases (3.8 %), the patients' place of residence was unknown and the distance to the hospital was marked as ‘not determined’. Patient age ranged from newborns to elderly (0–99 years; median = 31.8 years; mean = 37.1 ± 0.5 years). Those aged >65 received significantly more diagnoses per person (mean = 3.40) than the whole population (mean = 2.48) (*t*-test, t (880) = 10.12, p < 0.001). There was no significant variation in diagnoses per patient across sex, nationality, distance, or ethnicity (overall mean: 2.17; ANOVA: p = 0.146) **(Appendix –** Supplementary Table S1).

The most frequent reasons for visits were symptoms of a cold and/or sore throat related to acute nasopharyngitis, pharyngitis, or tonsillitis (Table 2). The next most frequent outcome was ‘no diagnosis,’ indicating that signs, symptoms, and available diagnostic procedures did not yield a final diagnosis. Other frequent diagnoses included routine antenatal visits, parasitic diseases, and non-communicable diseases such as type 2 diabetes and hypertension. Table 2 shows grouped hospital visit reasons with a frequency of ten or more, while those with fewer than ten visits are listed in **Appendix –** Supplementary Table S2.

**Table 2:** Most-frequently observed primary and grouped reasons for a hospital visit in the San Miguel patient population.

Primary reason for visit (frequency =>10)	Frequency n (%)
Acute symptoms of a cold or sore throat	144 (7.3 %)
*Acute nasopharyngitis*	*85 (4.3 %)*
*Acute pharyngitis*	*34 (1.7 %)*
*Acute tonsillitis*	*25 (1.3 %)*
No diagnosis	131 (6.6 %)
Supervision during pregnancy	114 (5.8 %)
*Normal pregnancy*	*80 (4.1 %)*
*High-risk pregnancy*	*20 (1.0 %)*
*Other,* e.g.*, labelled as pregnancy, childbirth or the puerperium; abdominal pregnancy*	*14 (0.7 %)*
(Intestinal) parasitic diseases defined by eosinophilia in the complete blood count	112 (5.7 %)
Type 2 diabetes mellitus	81 (4.1 %)
Hypertensive diseases	80 (4.1 %)
*Essential hypertension*	*78 (3.9 %)*
*Other (i.e., heat disease)*	*2 (0.1 %)*
Anemias or erythrocyte disorders	67 (3.4 %)
*Iron deficiency anemia*	*33 (1.7 %)*
*Other anemias, non-specified*	*33 (1.7 %)*
*Due to chronic disease*	*1 (0.1 %)*
Urinary tract infection	60 (3.0 %)
*Lower UTI: Cystitis*	*25 (1.3 %)*
*Other, non-specified*	*35 (1.8 %)*
Routine general health check-up	56 (2.8 %)
Gastritis	51 (2.6 %)
*Helicobacter pylori -*	*27 (1.4 %)*
*Helicobacter pylori +*	*21 (1.1 %)*
*Due to other causes*	*3 (0.2 %)*
Dyslipidemias	43 (2.2 %)
*Hypertriglyceridemia*	*20 (1.0 %)*
*Primary hypercholesterolemia*	*17 (0.9 %)*
*Secondary hypercholesterolemia*	*5 (0.3 %)*
*Mixed hyperlipidemia*	*1 (0.1 %)*
Gastroenteritis or colitis of infectious origin, i.e., symptoms of diarrhea	46 (2.3 %)
Localized abdominal pain	34 (1.7 %)
Lower respiratory tract infections	24 (1.2 %)
*Bronchitis*	*14 (0.7 %)*
*Pneumonia*	*10 (0.5 %)*
No pathology	22 (1.1 %)
Vulvovaginal candidiasis	20 (1.0 %)
Female pelvic inflammatory diseases	18 (0.9 %)
Lower back pain	16 (0.8 %)
Fever of unknown origin	16 (0.8 %)
Disruption of operation wound in most cases wound infection	16 (0.8 %)
Generalized abdominal pain	14 (0.7 %)
Obstipation	13 (0.7 %)
Obesity	13 (0.7 %)
Diabetic foot ulcer	12 (0.6 %)
Irritable bowel syndrome	12 (0.6 %)
Dizziness and giddiness without a known cause	12 (0.6 %)
Mycoses	11 (0.6 %)
Diabetic skin lesions	11 (0.6 %)
Intercostal pain	11 (0.6 %)
Sum	1260 (63.8 %)

Most frequent reasons for hospital visits were, when appropriate, grouped together with closely related diagnoses (e.g., anaemias other than iron deficiency anaemia). This table includes only reasons for hospital visits with a frequency greater than 10, representing a total of 1260 registered visit reasons. Reasons with lower frequencies (n = 715) are presented in Supplementary Table S2. For each diagnosis, the table reports both the absolute frequency and the corresponding percentage of all registered visit reasons (n = 1975).

The diagnoses in Table 2 and **Appendix -** Supplementary Table S2 were made in the outpatient clinic in 1908/1975 cases (97 %). A total of 67/1975 (3 %) diagnoses were made in emergency care, 19/67 (28 %) involving children and 48/67 (72 %) adults **(Appendix –** Supplementary Tables S3, S4, S5**).** The most frequent emergency diagnosis was gastroenteritis or colitis of infectious origin (3/19; 16 %). In adults, delivery was the most common emergency diagnosis (4/48; 8 %).

### 3.2 Diagnostic tools applied

The most frequently used laboratory tool at SMH was the complete blood count, used in 447/1755 cases (25 %) between November 2021 and February 2022 (Table 3). Laboratory tests are divided into five categories: clinical chemistry, hematology, microscopy, rapid tests, and agglutination tests. Clinical chemistry analysis by spectrophotometer was the most frequent, used in 717/1755 cases (41 %). A complete overview of laboratory tools used is in **Appendix –** Supplementary Table S6**.** Ultrasound was primarily used for diagnosing and examining pregnant women. Although an X-ray machine was present at the hospital, it had very limited capacity. It was used only five times during the examination period, primarily for pediatric cases, as children under ten accounted for 4/5 (80 %) of examinations. These included imaging of the lower extremities (2/5, 40 %), upper extremities (1/5, 20 %), and thorax (1/5, 20 %). The existing X-ray unit was small, produced low-resolution images, and was inadequate for imaging an adult thorax. For this reason, despite limited use reported in Table 3, an X-ray machine was classified as unavailable and needed (Fig. 1).

**Table 3:** The type and frequency of diagnostic tests used in San Miguel Hospital.

Type of diagnostic tests used Nov 2021–Feb 2022	Freq
Laboratory	1753
*Chemistry (Glucose, Cholesterol, ALAT/ASAT/Gamma GT, Creatin, Hb, Uric acid, Hb1Ac, Urea, Alkaline phosphatase, Bilirubin*	*715*
*Hematology (Complete blood count)*	*447*
*Microscopy (Urine - both microscopy and dipstick, Parasitology, Feces, Vaginal secretion, KOH)*	*357*
*Rapid tests (Dengue, H. Pylori, Pregnancy test, SARS-CoV-19, HIV, Syphilis, Malaria, occult blood test, Tuberculosis, Hepatitis B)*	*188*
*Agglutination (CRP, Blood type)*	*46*
Ultrasound	313
*Pregnancy ultrasound (abdominal or transvaginal based on gestational age)*	*132*
*Abdominal (convex)*	*93*
*Transvaginal*	*82*
*Other (linear)*	*6*
EKG	23
Radiography	5
*Lower extremity*	*2*
*Upper extremity*	*2*
*Thorax*	*1*

Pregnant women underwent either an abdominal or transvaginal ultrasound.

Knowing which diagnostic tools were applied, it is important to assess their role in confirming the final diagnosis. Table 4 shows diagnosis confirmation regardless of presentation timing (primary or follow-up consultation shown in **Appendix –** Supplementary Fig. S2**)**. Of the 1975 diagnoses, 1307 (66 %) were confirmed with existing tools, 466 (24 %) could not be confirmed, and for 202 (10 %), the final diagnosis was undetermined. In 931/1975 (47 %) diagnoses, adequate diagnostic tools were used, while in 288/1975 (15 %) diagnoses, tools were insufficient, meaning at least one diagnostic tool was missing. In 756/1975 (38 %) diagnoses, signs and symptoms alone were sufficient to reach a final diagnosis (e.g., dermatological lesions typical for eczema). Notably, 186/1975 (9 %) diagnoses could not be confirmed due to a lack of diagnostic tools. In 80/1975 (4 %) diagnoses, despite lacking adequate tools, a final diagnosis was reached, such as pelvic inflammatory disease (PID), where clinical presentation and C-reactive protein determination (a non-specific acute-phase protein indicating infection) were used to reach a diagnosis. The missing diagnostic tools in these cases could have ruled out specific diagnoses. In 931/1975 (47 %) diagnoses, adequate diagnostic tools were available, either confirming or ruling out diagnoses. Of these, 148/1975 (7 %) diagnoses could not be confirmed despite adequate tools. In 202/1975 (10 %) diagnoses, confirmation could not be achieved, either due to no final diagnosis or no pathology found. In 22/202 (11 %) of these cases (22/1975; 1 % of all diagnoses), this was due to lack of adequate diagnostics.

**Table 4:** Frequency of confirmation of diagnoses and application of adequate diagnostics in San Miguel Hospital.

-	Diagnosis confirmed N =	Diagnosis not confirmed N =	Diagnosis confirmed n.d. N =	Sum
Adequate diagnostics N =	672*/1975 = 34 %*	148*/1975 = 7 %*	111*/1975 = 6 %*	931*/1975 = 47 %*
No adequate diagnostics N =	80*/1975 = 4 %*	186*/1975 = 9 %*	22*/1975 = 1 %*	288*/1975 = 15 %*
Adequate diagnostics n.a. N =	555/*1975 = 28 %*	132/*1975 = 7 %*	69*/1975 = 3 %*	756*/1975 = 38 %*
Sum	1307/*1975 = 66 %*	466/*1975 = 24 %*	202/*1975 = 10 %*	1975 *100 %*

The diagnoses and adequate diagnostics were either confirmed, not confirmed or not determined (n.d.)/not applicable (n.a.). No differentiation between diagnoses from the primary and follow-up consultation was made. Diagnostic tools were labelled adequate when their method led to a confirmation of a diagnosis on an appropriate level of certainty.

### 3.3 Diagnostic tools missing

Non-confirmable essential final diagnoses—requiring additional diagnostics—include pneumonia, influenza, and pelvic inflammatory disease **(Appendix –** Supplementary Table S7**).** When no final diagnosis could be reached or confirmed, the lacking diagnostic tools were reported (**Appendix –** Supplementary Tables S8 and S9). Most frequently needed were bacterial cultures and sexually transmitted infection tests. Additionally, potassium hydroxide (KOH) microscopy - used to detect and differentiate dermatophyte fungal infections -and a higher-capacity X-ray machine—able to image an adult thorax—were required. These needs were reported regardless of whether a final diagnosis was confirmed and listed whenever a healthcare professional identified them as missing.

Multiple factors influence decisions on implementing a new diagnostic tool. Therefore, all tools were rated for accessibility based on criteria shown in Table 1. This included staff training, implementation costs, and the feasibility of collaborating with another health center. Rapid tests and microscopy were deemed easily accessible. Hematological and chemistry tests were rated easy to medium in the San Miguel Hospital context. Diagnostic tests like cervical smears available in nearby centers were also rated easy to medium. ELISAs used for thyroid function (TSH, T4) and prostate-specific antigen (PSA) testing had medium to difficult accessibility. PCR diagnostics were considered difficult due to cost, contamination risk, and staff training requirements. X-ray machines—costing over $100,000—were also classified as difficult to access and implement at SMH. Anatomical pathology was rated as easy-to-medium not because on-site implementation is simple, but because external collaboration with qualified pathologists was feasible and ultimately adopted, making access to the service achievable despite local constraints.

The local need and accessibility of each tool determined implementation potential, as shown in Fig. 1. Green-colored tools like KOH were both highly needed and easily implementable. Red and orange zones included tools that were hard to access, minimally needed, or both. For example, X-ray machines were expensive but essential, while computed tomography (CT) scans were costly, rare, and less needed. Bacterial cultures had lower costs and higher supply availability but required more expertise.

## 4 Discussion

The primary aim of this study was to evaluate the need and accessibility of diagnostic tools for disease diagnosis in the rural district of Putumayo, Ecuador, focusing on San Miguel Hospital (SMH). Our findings indicate that, despite its remote location and limited resources, SMH is relatively well-equipped to provide confirmed diagnoses. However, additional investments in diagnostic methods are necessary to optimize the diagnostic process in this low-resource setting. A systematic evaluation framework that considers local patient characteristics and disease prevalence has proven useful in identifying priority areas for improvement. This approach may serve as a valuable model for optimizing diagnostic capabilities in similar low-resource healthcare settings. In the local context of SMH, in 466 cases (24 % of all diagnoses), a final diagnosis could not be confirmed. In 148/466 (32 %) of those cases, this was despite availability of adequate diagnostic tools. This may have occurred in cases where extensive diagnostics were deemed unnecessary after ruling out more severe differential diagnoses using the available diagnostic tools. This also highlights the value of the diagnostic tools used in these cases, as they helped rule out more serious conditions despite often yielding negative results.

A similar situation could apply to those cases where a diagnosis was not confirmed, while no lack of diagnostic tools was reported either (132/466, 28 %): usage of diagnostic tools to confirm a final diagnose would not have been justified due to the mild clinical presentation and/or self-limiting character of the symptoms. When the confirmation of a final diagnosis was not determined, this was either because no pathology was found or because it remained unclear, based on the reporting whether or not a final diagnosis had been reached. This happened mostly in the early days after opening the hospital, when medical records were most susceptible to incomplete information or unspecific labelling of diagnoses. During the course of this study data entry into the medical records improved significantly.

Central to the method proposed here is the local patient population and the pathology presented by it, as these shape the healthcare demand. The demographics show that over 5 % (44/796) of the patients are either Kichwa, Shuar, Siona or of other ethnic indigenous background (**Appendix –** Supplementary Table S1). Given that indigenous communities are identified as vulnerable to inequality of healthcare utilization and are often located at greater distances from adequate healthcare and hospitals [10], [11], [12], the representation of indigenous persons in the first 800 patients of the SMH is promising. In addition, the numbers of individuals self-identifying as belonging to an indigenous community may be underestimated, potentially influenced by factors such as social and cultural dynamics [[15], [16], [17]]. Within this population, like in many other low-resource settings, a wide variety of diseases is seen in.

Table 2. Not only infectious diseases but also non-communicable diseases like diabetes and hypertension are frequently encountered. While the *Helicobacter pylori* induced gastritis found in this study is known for its high disease prevalence in Latin America, non-communicable diseases in the SMH patient population, such as diabetes and related lifestyle factors such as obesity, correspond to a more global pattern of increase in prevalence of non-communicable diseases in an aging population [18,19]. The other frequently encountered diagnosis in SMH – intestinal parasitic infestation with geohelminths – has been identified by previous studies as distinctive of rural Ecuador [20]. Anemia, historically found predominantly in rural areas in Ecuador, is now more evenly distributed between rural and urban populations (for women in the reproductive age) [21,22]. Our data shows that it remains prevalent within the population of inhabitants of the Amazon. Over time, if changes in the patient population occur, disease prevalence and presentation might change too, emphasizing the need for continuous registration and investigation of the local healthcare demands. Our data also shows that with a broad spectrum of clinical presentations, it is of importance for a hospital like SMH to have expertise on the design of primary care as well as emergency, and more complex care (**Appendix -** Supplementary Tables S2, S3, S4).

That expertise is already present in SMH is shown by the fact that, equipped with basic diagnostic tools and a staff of international and local healthcare workers, SMH is capable of diagnosing 66 % of all pathology presented by patients, at a development stage which has not reached the opening phase for inpatient care yet (Table 4). The frequent use of ultrasound—313 times during this study (Table 3)—reflects SMH's strong expertise in gynecological and obstetric care. This is further supported by the fact that no pregnant women appear in the data on missing diagnostics or non-confirmable diagnoses, indicating the hospital's ability to effectively care for this vulnerable population in the region [23].The diagnostic tools needed to confirm diagnoses were identified as possible areas of healthcare improvement and expansion in San Miguel Hospital in Fig. 1. The hospital already has most essential diagnostic tools in place, with few diagnostic tools identified in the green and accessible area. KOH preparations to diagnose fungal infections, and influenza and chikungunya rapid tests, would be valuable and feasible additions. Rapid tests are easy to perform and provide quick results, which is a strength in low-resource settings; however, they have limitations including relatively high detection limits and lower sensitivity compared to laboratory-based methods. While influenza rapid tests could increase the number of confirmed diagnoses, their clinical impact is limited because many patients could be managed effectively under the broader category of ‘influenza-like illness’ without specific antiviral treatment. Adding anatomical pathological services is thought to be of considerable value, despite the fact that implementation in a remote area would be challenging due to the costs of establishing such a service and a lack of both educated manpower and patient volumes. Providing this service externally is therefore a practical and more appealing option when available. For this reason, externally located pathologists were employed for the examination of smears at SMH after this study ended.

Like with the pathology examinations, the results of this study inspired immediate action on the implementation of the KOH diagnostic, explaining the need for KOH in Fig. 1, and documenting its use once in Appendix - Table S7. Although many fungal skin infections can be diagnosed clinically, KOH microscopy was prioritized at SMH to support clinician decision-making in atypical, recurrent, or treatment-resistant cases, providing low-cost confirmation and increasing diagnostic confidence in this low-resource setting. These direct actions taken by the hospital further emphasize the importance of collection and analysis of local data. The electronic record-keeping system of the hospital facilitated this data collection and analysis, which would have proven much more challenging with non-digital patient records. A basic format registering the patient history, physical examination, diagnostic results, diagnosis, treatment and overall patient characteristics was crucial for this study. The use of International Classification of Diseases (ICD) codes enabled data analysis and assisted physicians in their administration. Additionally, open text fields in the electronic patient files allowed physicians to note any additional considerations regarding diagnosis and diagnostics, enhancing data reliability.

This approach builds on the REASSURED framework by Land et al., emphasizing accessibility and the need for diagnostic tools in resource-limited settings [24]. By evaluating the availability and feasibility of existing diagnostics alongside local healthcare demands, we identify gaps where targeted investments could have the greatest impact. This methodology not only informs resource allocation but also supports optimized treatment strategies and improved patient outcomes in underserved regions. Notably, accessibility factors—such as affordability, user-friendliness, and ease of specimen collection, as assessed by local physicians—were included, whereas test specificity and sensitivity were not factored into the scoring, representing a study limitation.

This study has several limitations. First, data collection began at the hospital's inception, when electronic documentation was at its least developed, potentially affecting data completeness and accuracy. The retrospective nature of the analysis further complicated the identification of missing diagnoses, as certain diagnostic decisions were not always explicitly documented by physicians. Additionally, while Dutch clinical guidelines were used as a reference and adapted to the local context where necessary, this approach may have limited the specificity of the findings to the Ecuadorian healthcare setting.

Future research should prioritize larger, prospective studies conducted over an extended period to generate more robust evidence on diagnostic needs in low-resource settings. The integration of artificial intelligence or other advanced methodologies may enhance the efficiency and accuracy of diagnostic assessments. Moreover, expanding the evaluation framework to include additional REASSURED criteria, such as the sensitivity and specificity of diagnostic tests, could provide a more comprehensive assessment of available tools. Ultimately, such efforts may not only improve diagnostic capabilities in resource-limited settings but also direct the research into the development of new rapid diagnostic tests tailored to these environments**.**

## 5 Conclusions

Conclusively, the San Miguel Hospital has shown to provide adequate outpatient healthcare in the way that it is able to diagnose most of its patients and possesses the most-needed and easily accessible diagnostic tools for its specific patient population. We demonstrate here that healthcare improvements made through optimizing the availability of diagnostic tools are of value when based on regional data, seen that they inspired immediate action in the SMH. The method proposed here – when extrapolated and adapted to comparable health care situations – should thus provide a tool for anyone considering healthcare investments in a low resource, rural setting. For the Amazon basin specifically, larger, prospective studies should be performed over a longer period of time in order to provide more conclusive evidence on the needs of the population.

## CRediT authorship contribution statement

**Willemijn Johanna Catharina van Keizerswaard:** Writing – review & editing, Writing – original draft, Visualization, Formal analysis, Data curation. **Jacob van der Ende:** Writing – review & editing, Writing – original draft, Validation, Resources, Project administration, Formal analysis, Data curation. **Carolien Maria Bouwman:** Writing – review & editing, Formal analysis, Data curation. **Maria Vanessa Dávila Campos:** Writing – review & editing, Data curation. **Martin Peter Grobusch:** Writing – review & editing, Writing – original draft, Validation, Supervision, Resources, Project administration, Methodology, Formal analysis, Data curation, Conceptualization.

## Ethics statement

Ethical approval covering prospective and retrospective data collection for this project was granted by the ethical committee (Comité de Ética de Investigación de Seres Humanos (CEISH)) of the Universidad San Francisco de Quito on April 1st^,^ 2024 (Ref N. 061-2024-CA24015IN-CEISH-USFQ). The informed consent was waived by the ethics committee for all prospective and retrospective data.

## Consent for publication

Not applicable.

## Availability of data and materials

The datasets used and/or analyzed during the current study are available from the corresponding author on reasonable request.

## Funding sources

This work was supported by the authors, and in part covered by MPG's research funds at the Center of Tropical Medicine and Travel Medicine, 10.13039/100019573Amsterdam University Medical Centers.

## Declaration of competing interest

The authors declare that they have no known competing financial interests or personal relationships that could have appeared to influence the work reported in this paper.

## List of abbreviations

Abbreviation Definition.

CRP C-reactive protein.

CT Computed Tomography.

EMR(s) Electronic Medical Records.

GDP Gross Domestic Product.

ICD International Classification of Diseases.

KOH Potassium Hydroxide.

NHG Nederlands Huisartsen Genootschap (the Dutch National Guidelines for general practitioners).

PID Pelvic Inflammatory Disease.

PSA Prostate-Specific Antigen.

SMH San Miguel Hospital.

STI Sexually Transmitted Infections.

TSH Thyroid Stimulating Hormone.

T4 Thyroxine.

WBD Waterborne Diseases.
