# Supplementary material for: Optimizing health care delivery by adapting diagnostics in a low-resource setting: The case of San Miguel Hospital, Sucumbíos, Ecuador
Source: New Microbes New Infect. 2025 Dec 31;70:101696. doi: 10.1016/j.nmni.2025.101696 (PMC12856848; doi:10.1016/j.nmni.2025.101696)
Supplement: Multimedia component 1 [file mmc1.docx]

**Appendix A. Supplementary Data**

**Supplementary Table S1.** Patient demographics of San Miguel Hospital, Sucumbíos, Ecuador.

|  | Number of patients | % of all patients | Number of consultations | % of all consultations | Number of diagnoses | % of all diagnoses | Mean number of received diagnoses per patient | Mean number  of diagnoses  per  consultation |
| --- | --- | --- | --- | --- | --- | --- | --- | --- |
| Inhabiting country |  |  |  |  |  |  |  |  |
| Ecuador | 708 | 88.94 | 1523 | 92.30 | 1820 | 92.15 | 2.57 | 1.20 |
| Colombia | 83 | 10.43 | 122 | 7.39 | 150 | 7.59 | 1.81 | 1.23 |
| USA | 2 | 0.25 | 2 | 0.12 | 2 | 0.10 | 1.00 | 1.00 |
| n.d. | 3 | 0.38 | 3 | 0.18 | 3 | 0.15 | 1.00 | 1.00 |
| *Total* | *796* |  | *1650* |  | *1975* |  |  |  |
| Distance to the hospital (km) |  |  |  |  |  |  |  |  |
| <5 | 474 | 59.55 | 1100 | 66.67 | 1309 | 66.28 | 2.76 | 1.19 |
| 5-24 | 119 | 14.95 | 217 | 13.15 | 267 | 13.52 | 2.24 | 1.23 |
| 25-49 | 73 | 9.17 | 124 | 7.52 | 151 | 7.65 | 2.07 | 1.22 |
| 50-99 | 67 | 8.42 | 128 | 7.76 | 144 | 7.29 | 2.15 | 1.13 |
| >100 | 33 | 4.15 | 40 | 2.42 | 49 | 2.48 | 1.48 | 1.23 |
| n.d. | 30 | 3.77 | 41 | 2.48 | 55 | 2.78 | 1.83 | 1.34 |
| *Total* | *796* |  | *1650* |  | *1975* |  |  |  |
| Ethnicity |  |  |  |  |  |  |  |  |
| Mestizo | 714 | 89.70 | 1508 | 91.39 | 1794 | 90.84 | 2.51 | 1.19 |
| Kichwa | 39 | 4.90 | 63 | 3.82 | 80 | 4.05 | 2.05 | 1.27 |
| Afro Ecuatoriano | 15 | 1.88 | 35 | 2.12 | 49 | 2.48 | 3.27 | 1.40 |
| Siona | 2 | 0.25 | 4 | 0.24 | 4 | 0.20 | 2.00 | 1.00 |
| Shuar | 3 | 0.38 | 7 | 0.42 | 7 | 0.35 | 2.33 | 1.00 |
| Other | 9 | 1.13 | 10 | 0.61 | 12 | 0.61 | 1.33 | 1.20 |
| n.d. | 14 | 1.76 | 23 | 1.39 | 29 | 1.47 | 2.07 | 1.26 |
| *Total* | *796* |  | *1650* |  | *1975* |  |  |  |
| Age (years) |  |  |  |  |  |  |  |  |
| 0-5 | 97 | 12.19 | 218 | 13.21 | 270 | 13.67 | 2.78 | 1.24 |
| 6-18 | 136 | 17.09 | 248 | 15.03 | 280 | 14.18 | 2.06 | 1.13 |
| 19-65 | 477 | 59.92 | 948 | 57.45 | 1133 | 57.37 | 2.38 | 1.20 |
| >65 | 86 | 10.80 | 236 | 14.30 | 292 | 14.78 | 3.40 | 1.24 |
| *Total* | *796* |  | *1650* |  | *1975* |  |  |  |
| Sex |  |  |  |  |  |  |  |  |
| Female | 501 | 62.94 | 1066 | 64.61 | 1281 | 64.86 | 2.56 | 1.20 |
| Male | 295 | 37.06 | 584 | 35.39 | 694 | 35.14 | 2.35 | 1.19 |
| *Total* | *796* |  | *1650* |  | *1975* |  |  |  |

**Supplementary Table S2. Less-frequently observed primary reasons for a hospital visit in the San Miguel patient population.** The primary reasons for consultation with a frequency of <10 were partially grouped into major symptomatically related diseases and disorders such as diseases and symptoms of the skin. NB these groups do not include the most frequently observed primary reasons for a hospital visit in the San Miguel patient population shown in **Table 2**.

| **Primary reason for visit** | **Freq** |
| --- | --- |
| Diseases and symptoms of the skin | 121 |
| *Impetigo* | *9* |
| *Tinea pedis* | *9* |
| *Pyogenic abscess of the skin* | *8* |
| *Pyogenic bacterial infections of the skin or subcutaneous tissue* | *7* |
| *Bacterial cellulitis of skin or lymphangitis* | *5* |
| *Diseases of the skin* | *5* |
| *Non-pyogenic bacterial infections of the skin* | *4* |
| *Molluscum contagiosum (water warts)* | *4* |
| *Certain skin disorders attributable to bacterial infection* | *4* |
| *Dermatitis and eczema* | *4* |
| *Rash localized* | *4* |
| *Cutaneous Leishmaniasis* | *3* |
| *Scabies* | *3* |
| *Certain skin disorders attributable to viral infection* | *3* |
| *Rash generalized* | *3* |
| *Common warts* | *2* |
| *Tinea unguium* | *2* |
| *Dermatitis or eczema of the hands or feet* | *2* |
| *Psoriasis* | *2* |
| *Urticaria of unspecified type* | *2* |
| *Acute papular skin eruption* | *2* |
| *Acute desquamating skin eruption* | *2* |
| *Disorders of skin colour* | *2* |
| *Infantile napkin dermatoses* | *2* |
| *Dermatoses provoked by heat or electricity* | *2* |
| *Superficial bacterial folliculitis* | *1* |
| *Viral infections characterized by skin or mucous membrane lesions* | *1* |
| *Certain skin disorders attributable to infection or infestation* | *1* |
| *Viral exanthems* | *1* |
| *Certain skin disorders attributable to fungal infection* | *1* |
| *Seborrheic dermatitis* | *1* |
| *Lichen simplex or lichenification* | *1* |
| *Inflammatory erythema’s and other reactive inflammatory dermatoses* | *1* |
| *Pruritis* | *1* |
| *Disorders of hair* | *1* |
| *Alopecia or hair loss* | *1* |
| *Infantile seborrheic dermatitis* | *1* |
| *Drug eruptions* | *1* |
| *Changes of the skin caused by external factors* | *1* |
| *Pressure ulceration* | *1* |
| *Allergic contact dermatitis* | *1* |
| *Epidermoid cyst* | *1* |
| *Acrochordon or polyp* | *1* |
| *Lipoma* | *1* |
| *Pyogenic granuloma* | *1* |
| *Acute erythematous skin eruption* | *1* |
| *Acute urticarial skin eruption* | *1* |
| *Acute blistering skin eruption* | *1* |
| *Chronic excoriation skin disorder* | *1* |
| *Burns of external body surface* | *1* |
| *Candidiasis of skin or mucous membranes* | *1* |
| Gynaecological and menopausal diseases and symptoms excluding STDs | 51 |
| *Abnormal frequency of uterine bleeding* | *5* |
| *Abnormal volume of uterine bleeding* | *5* |
| *Enlarged endometrium* | *3* |
| *Acquired abnormalities of ovary* | *3* |
| *Other or unspecified ovarian cysts* | *3* |
| *Miome in the uterus* | *3* |
| *Noninflammatory disorders of the female genital tract* | *2* |
| *Abnormal uterine or vaginal bleeding* | *2* |
| *Menstrual cycle bleeding disorders* | *2* |
| *Amenorrhea* | *2* |
| *Non menstrual bleeding* | *2* |
| *Pain related to vulva, vagina or pelvic floor* | *2* |
| *Mastitis not associated with maternity* | *2* |
| *Polycystic ovary syndrome* | *2* |
| *Dyspareunia* | *1* |
| *Abnormal duration of uterine bleeding* | *1* |
| *Excessive menstruation with irregular cycle* | *1* |
| *Menopausal or certain specified perimenopausal disorders* | *1* |
| *Menopause* | *1* |
| *Excessive bleeding in the premenopausal period* | *1* |
| *Postmenopausal atrophic vaginitis* | *1* |
| *Female infertility* | *1* |
| *Secondary female infertility* | *1* |
| *Female pelvic pain associated with genital organs or menstrual cycle* | *1* |
| *Noncyclic pelvic pain* | *1* |
| *Symptoms related to the menopause* | *1* |
| *Special screening for examination for neoplasms of cervix* | *1* |
| Disease and symptoms related to ear, nose, throat (ENT) | 42 |
| *Otitis externa* | *9* |
| *Impacted cerumen* | *5* |
| *Otitis media* | *5* |
| *Chronic rhinitis, nasopharyngitis or pharyngitis* | *2* |
| *Foreign body in larynx* | *2* |
| *Streptococcal pharyngitis* | *1* |
| *Diseases of the ear or mastoid process* | *1* |
| *Diseases of the external ear* | *1* |
| *Otomycosis* | *1* |
| *Non-infectious inflammation of external ear* | *1* |
| *Chronic serous or mucoid otitis media* | *1* |
| *Suppurative otitis media* | *1* |
| *Acute vestibular syndrome* | *1* |
| *Presbycusis* | *1* |
| *Disorders of the ear, not elsewhere classified* | *1* |
| *Acute sinusitis* | *1* |
| *Acute laryngopharyngitis* | *1* |
| *Allergic rhinitis* | *1* |
| *Non-allergic rhinitis* | *1* |
| *Diseases of vocal cords or larynx, not elsewhere classified* | *1* |
| *Dysphonia* | *1* |
| *Epistaxis* | *1* |
| *Foreign body in the ear* | *1* |
| *Cyst or mucocele of the nose or paranasal tissue* | *1* |
| Neurological disorders and symptoms | 27 |
| *Parkinsonism* | *5* |
| *Headache* | *5* |
| *Headache disorders* | *4* |
| *Post-herpetic polyneuropathy* | *3* |
| *Benign positional paroxysmal vertigo* | *3* |
| *Migraine* | *2* |
| *Tingling fingers or feet or toes* | *1* |
| *Hemiplegia* | *1* |
| *Epilepsy or seizures* | *1* |
| *Migraine with aura* | *1* |
| *Mononeuropathy* | *1* |
| Non-traumatic musculoskeletal diseases and symptoms | 49 |
| *Osteoarthritis* | *9* |
| *Osteoarthritis of knee* | *6* |
| *Internal derangement of knee* | *6* |
| *Symptoms, signs or clinical findings of the musculoskeletal system* | *4* |
| *Joint pain* | *4* |
| *Limb pain* | *3* |
| *Thoracic spine pain* | *3* |
| *Osteoarthritis of hip* | *2* |
| *Impingement syndrome of shoulder* | *2* |
| *Chondropathies* | *2* |
| *Diseases of the musculoskeletal system or connective tissue* | *1* |
| *Arthropathies* | *1* |
| *Osteoarthritis of wrist and hand* | *1* |
| *Acquired deformities of fingers and toes* | *1* |
| *Disorders of synovium or tendon* | *1* |
| *Enthesopathies of the inferior extremities* | *1* |
| *Cervical spine pain* | *1* |
| *Stiffness of joint* | *1* |
| Gastroenterological diseases and symptoms | 28 |
| *Cholelithiasis* | *6* |
| *Gastro-oesophageal reflux disease* | *4* |
| *Abscess of liver* | *4* |
| *Functional constipation* | *4* |
| *Gastritis due to external causes* | *3* |
| *Diseases of oesophagus* | *1* |
| *Obstruction of small intestine* | *1* |
| *Cholecystitis* | *1* |
| *Nausea and vomiting* | *1* |
| *Dyspepsia* | *1* |
| *Gastro-intestinal haemorrhage* | *1* |
| *Appendicitis* | *1* |
| Musculoskeletal trauma | 26 |
| *Thoracic contusion* | *9* |
| *Injuries to the knee or lower leg* | *4* |
| *Injuries to the hip or thigh* | *2* |
| *Laceration of the head without foreign body* | *2* |
| *Fracture of the distal epiphysis of the radius* | *2* |
| *Traumatism of the ankle or foot* | *1* |
| *Laceration of the head with a foreign body* | *1* |
| *Muscular distension or spraining* | *1* |
| *Injuries to the elbow or forearm* | *1* |
| *Injuries to the wrist or hand* | *1* |
| *Traumatism of the ankle or foot* | *1* |
| *Contusion of a toe* | *1* |
| Specified helminth diseases | 24 |
| *Strongyloidiasis* | *10* |
| *Ascariasis* | *7* |
| *Amoebiasis* | *4* |
| *Enterobiasis* | *1* |
| *Trichuriasis* | *1* |
| *Giardiasis* | *1* |
| Diseases and symptoms related to the eye | 22 |
| *Foreign body on external eye* | *4* |
| *Keratoconjunctivitis seca* | *4* |
| *Chalazion* | *3* |
| *Conjunctivitis* | *3* |
| *Pterygium* | *2* |
| *Blindness, binocular* | *1* |
| *Moderate vision impairment* | *1* |
| *Age-related cataract* | *1* |
| *Mucopurulent conjunctivitis* | *1* |
| *Infectious disorders of the eyelid* | *1* |
| *Trauma of the eye or orbita* | *1* |
| Diseases and symptoms related to the urinary tract excluding urinary tract infections | 21 |
| *Diseases of the urinary system, not specified elsewhere* | *4* |
| *Urolithiasis* | *4* |
| *Hydronephrosis with ureteral obstruction* | *2* |
| *Certain specified diseases of the urinary system* | *2* |
| *Acute pyelonephritis* | *1* |
| *Kidney failure* | *1* |
| *Acute kidney failure* | *1* |
| *Calculus of kidney* | *1* |
| *Certain specified disorders of kidney or ureter* | *1* |
| *Symptoms, signs or clinical findings involving the urinary system* | *1* |
| *Retention of urine* | *1* |
| *Dysuria* | *1* |
| *Polyuria* | *1* |
| Medical examination only | 21 |
| *Laboratory examination* | *9* |
| *General child health examination* | *7* |
| *Blood pressure control* | *2* |
| *General adult medical examination* | *1* |
| *Special screening examination for infectious diseases* | *1* |
| *Contact with health services for counselling* | *1* |
| Contraceptive related consultations | 25 |
| *Removal of contraception device: implant* | *7* |
| *Consultation for contraceptive device: implant placement* | *6* |
| *Pregnancy test* | *4* |
| *Vigilance of contraceptive medication: injection of contraceptive* | *3* |
| *Removal of intrauterine device* | *3* |
| *Informative consultation regarding contraceptives* | *1* |
| *Placement of intrauterine device* | *1* |
| Cardiac symptoms and diseases | 17 |
| *Tachycardia* | *5* |
| *Angina pectoris* | *4* |
| *Congestive cardiac insufficiency* | *3* |
| *Heart failure* | *1* |
| *Atrial fibrillation* | *1* |
| *Cardiac arrhythmia* | *1* |
| *Diseases of coronary artery* | *1* |
| *Cardiac murmurs and other cardiac sounds* | *1* |
| Sexually transmittable infections and related symptoms | 16 |
| *Vaginitis* | *6* |
| *Predominantly sexually transmitted infections* | *4* |
| *Trichomoniasis* | *2* |
| *Anogenital herpes simplex infection* | *1* |
| *Sexually transmittable infections, women* | *1* |
| *Vulvovaginal ulceration and inflammation* | *1* |
| *Gardnerella vaginosis* | *1* |
| Pregnancy complications | 13 |
| *Haemorrhage in early pregnancy* | *3* |
| *Excessive vomiting in pregnancy* | *2* |
| *Oligohydramnios* | *2* |
| *Complications following abortion, ectopic or molar pregnancy* | *1* |
| *Infections of bladder in pregnancy* | *1* |
| *Foetus or new-born affected by maternal factors or by complications of pregnancy, labour or delivery* | *1* |
| *Foetus or new-born affected by maternal complications of pregnancy* | *1* |
| *Disruption of obstetric wound* | *1* |
| *Non purulent mastitis associated with childbirth* | *1* |
| COVID-19 | 10 |
| Abortion | 9 |
| *Spontaneous abortion* | *4* |
| *Spontaneous abortion, incomplete without complications* | *3* |
| *Spontaneous abortion, incomplete with complications* | *1* |
| *Missed abortion* | *1* |
| Influenza | 9 |
| Throat or chest pain | 8 |
| Tension-type headache | 8 |
| Hyperplasia of prostate | 7 |
| Disorders of the breast | 7 |
| Mental symptoms and disorders | 6 |
| *Suicide attempt* | *2* |
| *Irritability* | *1* |
| *Depressive disorder* | *1* |
| *Anxiety or fear-related disorder* | *1* |
| *Disorders specifically associated with stress* | *1* |
| Tiredness | 6 |
| Delivery | 5 |
| Varicella zoster virus infection | 5 |
| Calcaneal spur | 5 |
| Chronic kidney disease | 5 |
| Decreased white blood cell count | 4 |
| Undernutrition | 4 |
| Asthma | 3 |
| Oral aphthae or aphthae-like ulcerations | 3 |
| Direct infections of joint | 3 |
| Chronic prostatitis | 3 |
| Sciatica | 3 |
| Decreased appetite | 3 |
| Injury, poisoning or certain other consequences of external causes | 3 |
| Posttraumatic wound infection | 3 |
| Allergic or hypersensitivity conditions | 3 |
| Allergic or hypersensitivity disorders involving the respiratory tract | 3 |
| Overweight or obesity in children <18 years | 5 |
| Neuropathic pain | 3 |
| Hernia | 3 |
| *Inguinal* | *1* |
| *Femoral* | *1* |
| *Umbilical* | *1* |
| Sleeping disorders | 2 |
| *Insomnia disorders* | *1* |
| *Nightmare disorder* | *1* |
| Anaphylaxis | 2 |
| *Drug-induced anaphylaxis* | *1* |
| *Anaphylaxis due to contact with allergens* | *1* |
| Candidiasis of lips or oral mucous membranes | 2 |
| Upper respiratory tract disorders | 2 |
| Mouth ulcers | 2 |
| Slow transit constipation | 2 |
| Surgical wound without complications | 2 |
| Hydrocele or spermatocele | 2 |
| Erectile dysfunction | 2 |
| Rheumatoid arthritis | 2 |
| Benign vascular neoplasms in children | 2 |
| Testicular pain | 2 |
| Localized oedema | 2 |
| Seizure due to acute causes | 2 |
| Gas gangrene | 1 |
| Dengue | 1 |
| Other viral diseases | 1 |
| Lower limb varicose veins | 1 |
| Diseases of the respiratory system | 1 |
| Allergic asthma with exacerbation | 1 |
| Lung infections, not specified otherwise | 1 |
| Cysts of oral or facial-neck region | 1 |
| Obstruction of large intestine | 1 |
| Certain non-infectious colitis or proctitis | 1 |
| Haemorrhoids or perianal venous conditions | 1 |
| Haemorrhoids | 1 |
| Abscess of anal region | 1 |
| Functional bloating | 1 |
| Infections of nail or perionychium | 1 |
| In growing nail | 1 |
| Gout | 1 |
| Residual foreign body in soft tissue | 1 |
| Myalgia | 1 |
| Osteoporosis | 1 |
| Diseases of prostate | 1 |
| Benign breast disease | 1 |
| Certain conditions originating in the perinatal period | 1 |
| Enlarged lymph nodes | 1 |
| Localized lymph node enlargement | 1 |
| Elevated white blood cell count | 1 |
| Decreased libido | 1 |
| Abnormal blood pressure | 1 |
| Abdominal tenderness | 1 |
| Intra-abdominal or pelvic swelling, mass or lump | 1 |
| Subcutaneous swelling, mass or lump | 1 |
| Symptom or complain of body part | 1 |
| Syncope and collapse | 1 |
| Injuries to the abdomen, lower back, lumbar spine or pelvis | 1 |
| Foreign body in alimentary tract | 1 |
| Burns of eye or internal organs | 1 |
| Harmful effects of drugs, medicaments or biological substances | 1 |
| Heat syncope | 1 |
| Immune system disorders involving white cell lineages | 1 |
| Hypoglycaemia without coma | 1 |
| Impaired glucose tolerance | 1 |
| Underweight in infants, children or adolescents | 1 |
| Calcium deficiency | 1 |
| Dehydration | 1 |
| Malaria, parasite not specified | 1 |
| **Total** | **715** |

**Table S3.** The causes of paediatric emergencies and the frequency of their occurrence.

| Diagnoses – paediatric patients | Freq |
| --- | --- |
| Gastroenteritis or colitis of infectious origin | 3 |
| Injury, poisoning or certain other consequences of external causes | 2 |
| Laceration of the head without foreign body | 2 |
| Ascariasis | 1 |
| Acute tonsillitis | 1 |
| Bronchitis | 1 |
| Generalized abdominal pain | 1 |
| Fever of unknown origin | 1 |
| Thoracic contusion | 1 |
| Fracture of the distal epiphysis of the radius | 1 |
| Foreign body in alimentary tract | 1 |
| Anaemias or other erythrocyte disorders | 1 |
| Allergic or hypersensitivity disorders involving the respiratory tract | 1 |
| Anaphylaxis due to contact with allergens | 1 |
| Undernutrition | 1 |

Data from patient consultations between the period of Nov 2021 and Feb 2022 in San Miguel Hospital.

**Table S4.** The causes of emergencies in adults and the frequency of their occurrence.

| Diagnoses – adult patients | Freq |
| --- | --- |
| Delivery | 4 |
| COVID-19 | 3 |
| Localized abdominal pain | 3 |
| Thoracic contusion | 3 |
| Gastroenteritis or colitis of infectious origin | 2 |
| Pneumonia | 2 |
| Spontaneous abortion | 2 |
| Supervision of normal pregnancy | 2 |
| COVID-19 | 2 |
| Pyogenic abscess of the skin | 1 |
| Gas gangrene | 1 |
| Atrial fibrillation | 1 |
| Acute laryngopharyngitis | 1 |
| Allergic asthma with exacerbation | 1 |
| Gastritis | 1 |

Data from patient consultations between the period of Nov 2021and Feb 2022 in San Miguel Hospital.

**Table S5.** The causes of emergencies in adults with a frequency of one.

| Other diagnoses of emergencies – adult patients (freq = 1) |
| --- |
| Abnormal volume of uterine bleeding, Kidney failure, Complications following abortion, ectopic or molar pregnancy, Haemorrhage in early pregnancy, Single spontaneous delivery head (outside of office hours), Suicide attempt, Throat or chest pain, Retention of urine, Fever of unknown origin, Injury, poisoning or certain other consequences of external causes, Foreign body in larynx, Anaemias or other erythrocyte disorders, Anaemia due to chronic disease, Drug-induced anaphylaxis, Epilepsy or seizures, Seizure due to acute causes, Laceration of the head with a foreign body. |

Data from patient consultations between Nov 2021 and Feb 2022 in San Miguel Hospital.

**Table S6.** Frequency of laboratory and chemistry tests used in San Miguel Hospital.

| Diagnostics used | Freq |
| --- | --- |
| Complete blood count Flowcytometry | 447 |
| Glucose  Point-of-care (POC) testing | 274 |
| Urine  Dipstick or/and microscopy | 251 |
| Cholesterol  Clinical chemistry - photometry | 143 |
| Parasitology  Microscopy (stool) | 100 |
| ALAT/ASAT  Clinical chemistry - photometry | 90 |
| Creatinine  Clinical chemistry – photometry | 86 |
| Helicobacter Pylori  Rapid test – chromatography | 47 |
| C Reactive Protein (CRP)  Agglutination test | 42 |
| Dengue  Rapid test – chromatography | 38 |
| Hb  Clinical chemistry - photometry | 38 |
| Pregnancy test  Rapid test – chromatography | 32 |
| Uric acid  Clinical chemistry -photometry | 32 |
| HbA1c  Clinical chemistry -photometry | 27 |
| SARS-CoV-19  Rapid test – chromatography | 26 |
| HIV  Rapid test – chromatography | 12 |
| Urea  Clinical chemistry - photometry | 12 |
| Syphilis  Rapid test – chromatography | 11 |
| Malaria  Rapid test – chromatography | 9 |
| Blood uric acid  Clinical chemistry - photometry | 7 |
| Occult Blood Test  Guaiac fecal blood test (FOBT) | 6 |
| Blood type  Agglutination test | 4 |
| Tuberculosis  Rapid test – chromatography | 4 |
| Alkaline phosphatase  Clinical chemistry -photometry | 3 |
| Bilirubin (direct/indirect/total)  Clinical chemistry -photometry | 3 |
| Hepatitis B  Rapid test – chromatography | 3 |
| Feces  Microscopy | 2 |
| Microscopy vaginal secretion | 2 |
| KOH  Microscopy | 1 |
| Microscopy otherwise | 1 |
| **Total** | **1753** |

**Table S7.** Top-ten diagnoses not confirmed / not further specifiable due to lack of diagnostic tool(s).

| Diagnosis not confirmed due to lacking diagnostics | Freq |
| --- | --- |
| Pneumonia | 10 |
| Influenza | 9 |
| Female pelvic inflammatory diseases | 9 |
| Fever of unknown origin | 8 |
| Unable to reach a final diagnosis | 7 |
| Localised abdominal pain | 6 |
| Gastroenteritis or colitis of infectious origin | 5 |
| Mycoses | 5 |
| Osteoarthritis | 5 |
| Vaginitis | 5 |

**Table S8.** Diagnostic tool(s) reported as missing in San Miguel Hospital and the frequency of reporting.

| Diagnostics missing | Freq |
| --- | --- |
| (Bacterial) cultures  (+Fungal culture) | 29  (+1) |
| STI tests  (Chlamydia antigen test) | 29  (+1) |
| KOH test | 24 |
| X-thorax | 20 |
| Influenza rapid test | 17 |
| Chikungunya rapid test | 16 |
| CT scan  (+ Angiography) | 12  (+1) |
| Pathology  Leishmaniasis | 9  (+6) |
| Thyroid tests: thyroid-stimulating hormone (TSH) and free thyroxine (T4) | 8 |
| Lung function tests | 4 |
| X-ray other than thoracic | 4 |
| PCR Herpes | 2 |
| Diagnostic laparoscopy | 2 |

Data from patient consultations between Nov 2021 and Feb 2022

**Table S9.** Diagnostic tool(s) identified as missing only once in all consultations.

Data from patient consultations between Nov 2021 and Feb 2022 in San Miguel Hospital.

| Other diagnostics missing (freq = 1) |
| --- |
| Albumin urine test, Amoeba serology, bHCG quantitative analysis, Cervical smear, coloscopy, EBV serology, Enterovirus PCR, Ferritin/B12/folic acid, Gastroscopy, Troponin rapid tests, Laryngoscopy, *Mycobacterium leprae* microscopy test, Mammography, PCR for Molluscum contagiosum, PSA ELISA, PT/APTT agglutination test, Rhinoscopy, Toxicology, Trematode serology, Histology, other viral PCR (2x), Tonometry |

**Figure S1.** Location of San Miguel Hospital and its patient population.


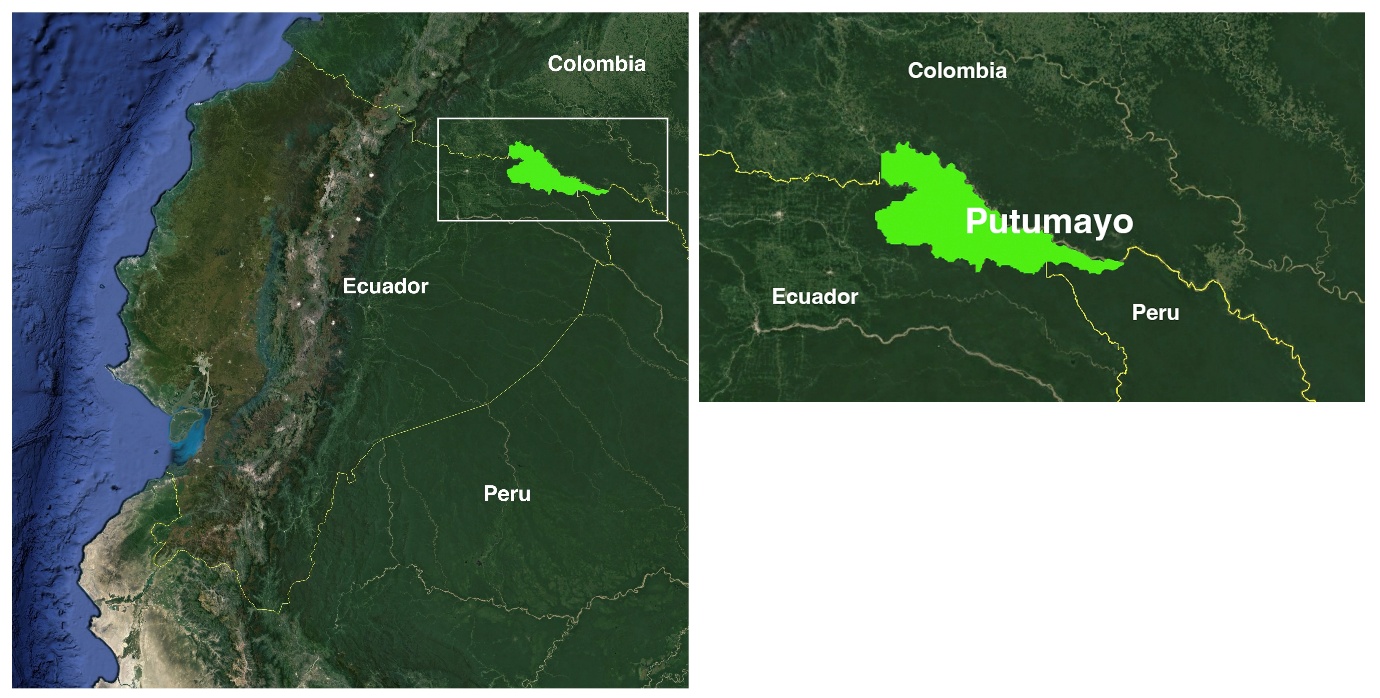


**Figure S2. Data extraction from the electronic medical records.** The electronic medical records of 796 patients visiting San Miguel Hospital, Sucumbíos, Ecuador were included in the study, accounting for a total number of 1650 consultations and 2003 final diagnoses. A total of 28 diagnoses were excluded due to a lack of sufficient data. Of the remaining diagnoses, 1534 were diagnosed in the primary visit. A total of 441 diagnoses was derived from a follow-up consultation. Diagnoses are classified as confirmed, not confirmed or not determined (n.d.).

Number of consultations

N = 1650

Primary consultation diagnosis

N = 1534

Follow-up consultation diagnosis

N = 441

Total number of diagnoses

N = 1975

Number of patients visiting San Miguel (Nov 2021 – Feb 2022)

N = 796

Diagnosis confirmed

N = 956

Diagnosis not

confirmed

N = 402

Diagnosis confirmed

N = 351

Diagnosis not confirmed

N = 64

Diagnosis confirmed n.d.

N = 176

Diagnosis confirmed n.d.

N = 26

Diagnoses excluded due to insufficient data N = 28
